# A Simulated Environment Experiment on Annoyance Due to Combined Road Traffic and Industrial Noises

**DOI:** 10.3390/ijerph120708413

**Published:** 2015-07-21

**Authors:** Catherine Marquis-Favre, Julien Morel

**Affiliations:** Univ Lyon, Ecole Nationale des Travaux Publics de l’Etat, Laboratoire Génie Civil et Bâtiment, 3 rue Maurice Audin, F-69518 Vaulx-en-Velin, France

**Keywords:** total annoyance, simulated environment, acoustical factors, non-acoustical factors, total annoyance model, combined noises, industrial noise, road traffic noise

## Abstract

Total annoyance due to combined noises is still difficult to predict adequately. This scientific gap is an obstacle for noise action planning, especially in urban areas where inhabitants are usually exposed to high noise levels from multiple sources. In this context, this work aims to highlight potential to enhance the prediction of total annoyance. The work is based on a simulated environment experiment where participants performed activities in a living room while exposed to combined road traffic and industrial noises. The first objective of the experiment presented in this paper was to gain further understanding of the effects on annoyance of some acoustical factors, non-acoustical factors and potential interactions between the combined noise sources. The second one was to assess total annoyance models constructed from the data collected during the experiment and tested using data gathered *in situ*. The results obtained in this work highlighted the superiority of perceptual models. In particular, perceptual models with an interaction term seemed to be the best predictors for the two combined noise sources under study, even with high differences in sound pressure level. Thus, these results reinforced the need to focus on perceptual models and to improve the prediction of partial annoyances.

## 1. Introduction

Environmental noise remains a major concern. For instance, a recent French survey showed that more than 86% of French people are annoyed by noise at home [1]. Annoyance is one of the five health effects that the World Health Organization (WHO) considered in order to estimate the burden of disease due to environmental noise [2]. The densification of urban areas, activities generated by economic growth and motorized transport are some of the driving forces for environmental noise exposure and its health effects. Moreover, these driving forces lead to a multiplication of various combined noise exposure situations. For these situations, there is a lack of regulatory framework. Actually, the environmental noise directive [3], obliging the production of noise maps for European agglomerations, only considers noise sources separately, thus neglecting combined noise exposure. However when it comes to noise action planning, especially in urban areas, public authorities have to deal with multiple noise sources.

Annoyance due to combined noises has been investigated by researchers for more than 40 years [4]. However, knowledge remains incomplete in terms of: (1) assessment of potential interactions between combined noises and of (2) total annoyance prediction to better take into account these interactions [5,6,7]. Such knowledge is essential for planning effective and efficient noise control and abatement strategies for combined exposure situations [8].

From a methodological point of view, noise annoyance may be studied either *in situ* or under laboratory conditions. Laboratory studies are related to short-term annoyance assessed in an imaginary situation for short noise sequences, thus the corresponding judgments represent an annoyance potential [9]. *In situ* studies are related to long-term annoyance and the collected responses reflect actual annoyance as respondents are asked at their home to give a retrospective judgment [10]. Laboratory and *in situ* studies have their own advantages [11] and appear complementary: Laboratory conditions allow to mainly investigate the different acoustical factors (e.g., sound level, spectral content, *etc.*) and their potential interactions when combined noises are considered (e.g., [7]); *in situ* conditions allow the study of non-acoustical factors (e.g., noise sensitivity, fear of danger from the noise source, *etc.*, see [5,12,13] for reviews) encountered in real-life situations. Simulated environment experiments, either in laboratory (e.g., [14,15]) or in field (e.g., [16]), allow one to approach real-life situations when investigating the influence on annoyance of acoustical and non-acoustical factors under controlled conditions: Participants are placed in a room furnished like a typical living room, and they can perform activities while exposed to noise sequences. For example, the simulated environment is useful to study the influence of some non-acoustical factors on annoyance under controlled conditions such as the type of activity carried out during the noise exposure and its disturbance which may be more difficult to assess through surveys (e.g., [17]).

This paper intends to contribute to a better characterization of annoyance due to combined road traffic and industrial noises through a simulated environment experiment. For the combined noise exposures studied, the aims of the experiment presented in this paper were: (1) to investigate potential effects on annoyance of some acoustical factors, non-acoustical factors, and of potential interactions between the combined noise sources, (2) to assess total annoyance models from literature in terms of their goodness-of-fit (models were constructed from the data collected during the experiment) and in terms of their predictive power using *in situ* data gathered during a socio-acoustic survey [18,19,20].

First the experimental method is presented (Section 2). The investigation of potential effects on annoyance and of potential interactions between combined noises is then detailed (Section 3). Section 4 deals with the assessment of total annoyance models both in terms of goodness-of-fit and of predictive power. The results are then discussed (Section 5) and the main conclusions are drawn (Section 6).

## 2. Experimental Method

### 2.1. Noise Recording

The road traffic noise and the industrial noise under study corresponded to noise exposure in a residential neighborhood of a suburban city of Lyon. The inhabitants of this neighborhood lived close to a pharmaceutical plant and were respondents of an *in situ* survey carried out on total annoyance due to combined road traffic and industrial noises [18,19,20]. The survey area was located across from the industrial plant and delimited by two open roads. The industrial plant emitted a continuous and stationary industrial noise composed of cooling and air blowing device noises. At peak hours of road traffic, the industrial noise was mainly masked by the urban road traffic noise, whereas at off-peak hours without road traffic noise events, the continuous and steady industrial noise was unmasked.

All the noise recordings were made using the ORTF technique (Schoeps MSTC 64 microphones and BBG windfields), and stored on a portable recorder (TASCAM HD-P2, sampled at 44.1 kHz with an amplitude resolution of 24 bits). The ORTF couple was placed at a height of 1.5 m and at least at 2 m from any reflecting wall [21].

Recordings of vehicle pass-by noises were undertaken close to traffic lights along open roads including the ones along the survey area. The axis of the ORTF couple was placed perpendicularly to the road [22]. The recorded pass-by noises corresponded to various urban vehicle types (light vehicles, heavy vehicles, powered-two-wheelers and buses) in various driving conditions (acceleration, deceleration, constant speed). The recordings were carried out during the day. From these recordings, road vehicle pass-by noises with no masking noise were considered in order to be used for stimuli construction (*cf.* Section 2.2). The duration of the pass-by noises ranged from 3 s to 9 s.

For recording the industrial noise, the ORTF couple was placed in front of a house located in the surveyed area and facing the main noise sources of the industrial plant (cooling and air blowing devices [20]). The recording was carried out at night to be free of masking environmental noise.

### 2.2. Stimuli

Noise sequences of 7 min 30 s were constructed from the recorded noises. This duration was chosen considering similar simulated environment experiments previously published (e.g., [15,16]).

The industrial noise sequence was constructed using the recorded continuous and steady industrial noise. In order to allow the results of this current study to be tested with the data collected *in situ* [18,19,20], the industrial noise exposure level observed during the survey was considered. The A-weighted equivalent sound pressure level of the industrial noise, denoted by L_ind_, was set in order to expose the experiment participants to indoor L_ind_ varying from 38 to 44 dB (A) in 2-dB (A) steps.

The road traffic noise sequence was constructed using the different recorded vehicle pass-by noises. This sequence had the following characteristics:

- Differences in A-weighted equivalent sound pressure level were considered between the vehicle pass-by noises that composed the road traffic noise sequence; they corresponded to mean differences observed *in situ* (*cf*. [23,24]) according to urban vehicle type (light vehicles, heavy vehicles, powered-two-wheelers and buses) and the driving conditions (acceleration, deceleration, constant speed);
- The considered composition of the urban road traffic corresponded to a traffic composition observed at peak hour (63% of light vehicles, 11% of heavy vehicles, 4% of buses, 22% of powered-two-wheelers);
- For the same reason as the one expressed for the industrial noise sequence, the A-weighted equivalent sound pressure level of the constructed road traffic noise sequence, denoted by L_road_, was set in order to expose the experiment participants to indoor L_road_ varying from 44 to 53 dB (A) in 3-dB (A) steps.

The construction of combined road traffic and industrial noise sequences was based on the principle advised by Berglund and Nilsson [25] in order to study annoyance due to combined noises. The stimuli consisted of all possible combinations of the four noise levels of the road traffic noise sequence with the four noise levels of the industrial noise sequence (*i.e.*, a 4 × 4 matrix). Thus, the experiment involved 16 stimuli: 4 “Road traffic noise equivalent sound pressure level—L_road_” × 4 “Industrial noise equivalent sound pressure level—L_ind_”.

**Figure 1:**
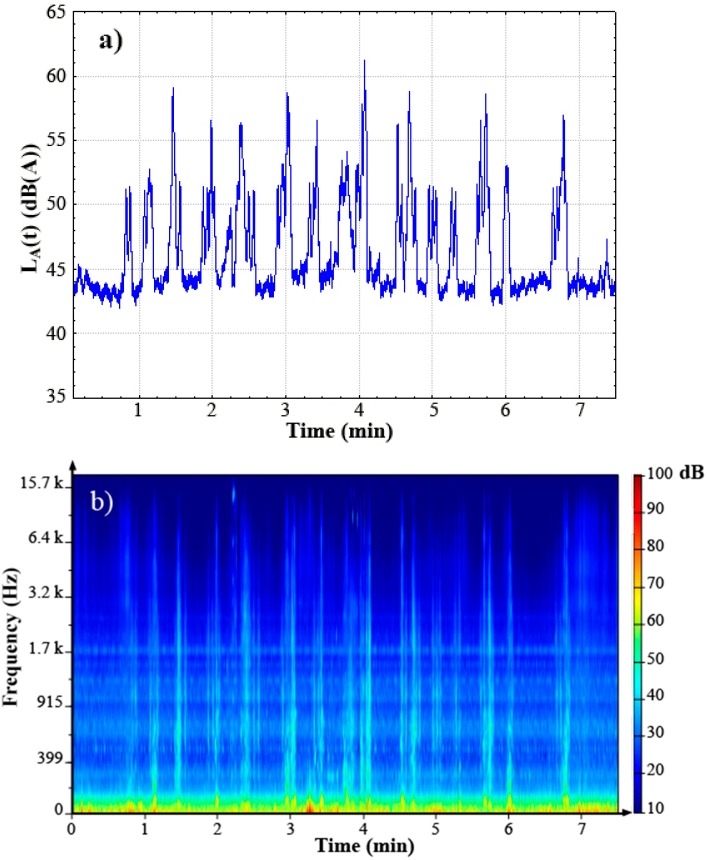
(**a**) A-weighted sound pressure level L_A_(t) *versus* time for the noise sequence combining the industrial noise set at 44 dB (A) with the road traffic noise set at 47 dB (A); (**b**) The spectrogram for the noise sequence combining the industrial noise set at 44 dB (A) with the road traffic noise set at 47 dB (A).

Figure 1(a) shows the A-weighted sound pressure level L_A_(t) *versus* time for the combined noise sequence with industrial noise L_ind_ set at 44 dB (A) and road traffic noise L_road_ set at 47 dB (A). It displays peaks in L_A_ (t) due to the road vehicle pass-bys.

Figure 1(b) presents the frequency content of the same noise sequence. It displays the energy content of the steady industrial noise distributed over different stationary components which are illustrated by horizontal lines (*i.e.*, same energy content *versus* time):

- At low frequencies (energy content for frequencies up to 400 Hz),
- At middle frequencies (energy content for frequencies ranged from 500 Hz to 800 Hz),
- At higher frequencies (energy content for frequencies ranged from 900 Hz to 1500 Hz),
- Two narrower components around 1.7 kHz and 2.3 kHz respectively.

More details of this frequency content are displayed in Supplementary Figure S1. The energy content due to the different road vehicle pass-bys within the sequence was illustrated by vertical lines (*i.e.*, energy content for a large frequency range and only present during the vehicle pass-bys). This energy content is often distributed from 80 Hz up to frequencies higher than 6.4 kHz, depending on the vehicle type and on its driving condition. Such sound frequency content due to urban road vehicle pass-by may be seen in Supplementary Figure S2.

During 57% of the combined noise sequence duration, the industrial noise was heard alone. During the other 43% of the sequence duration, the road traffic noise composed of different urban vehicle pass-bys masked the industrial noise. This was in agreement with the observation made *in situ* [18,19,20].

### 2.3. Experimental Setting

The experiment took place in a detached house located on the campus site of Vaulx-en-Velin, a suburban city of Lyon. The detached house was surrounded by a garden with trees and tall hedges. A residential road was in the vicinity at about 200 m. The residential urban sound environment around the detached house was rather quiet (percentile level L_90_ = 39 dB (A) during the day period). There would be human activities at certain periods of the day or wind in the tree leaves resulting in a higher outdoor background noise. Thus, attention was paid to carry out the experiment at quiet hours towards human activities and with appropriate weather conditions. During the experiment the window was kept partially opened, and the indoor A-weighted equivalent sound pressure level of the background noise in this condition was measured at 36 dB (A) (indoor percentile level L_90_=32 dB (A)). For such condition, the industrial noise composed of cooling and air blowing device noises was always clearly distinguishable as it was the case in the survey area at periods of the day when no urban road vehicles were passing-by [18,19,20].

Two rooms with view on the garden and its hedges were used for the experiment. One room was furnished like a conventional living room and dedicated to hosting the participants during the experiment. The other room, adjacent to the first one, was dedicated to the control of the experiment.

**Figure 2:**
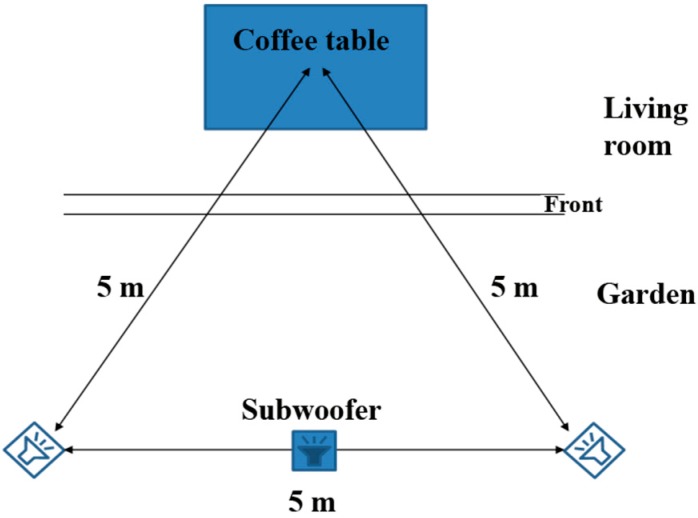
Experimental setting.

The experimenter, placed in the control room, reproduced the stimuli in random order, using SoundForge^©^Sony software. The 2.1 reproduction system (2 Dynaudio Acoustics BM5A Active loudspeakers associated to a Dynaudio Acoustics BM9S subwoofer) was placed outside the living room in the garden as shown in Figure 2. The participants were seated on sofas around the coffee table. The central point between the participants was approximately the center of the coffee table. This central point faced the loudspeakers placed at a 1 m 20 height, forming an equilateral triangle of 5 m edge in agreement with Beck and Zacharov’s recommendations [26].

As the noise sequences were reproduced from outside to inside the living room with window partially-open, a frequency filtering was taken into account in the noise exposure of participants. The reproduced noise sequences were recorded inside at the central point to check the stimuli indoor levels defined in Section 2.2.

### 2.4. Procedure

Due to the experiment duration (3 h), the experiment was performed on two separate days. During each part (of about 1 h 30 each) participants were submitted to eight of the 16 noise sequences. Furthermore, four stimuli with combined road traffic and industrial noises were added in each part of the experiment in order to assess the homogeneous character of responses from one part of the experiment to the other, when the same stimuli were considered. First, participants had to read a paper with general instructions:

- They were told that they would be exposed to different sound environment sequences comprising industrial and road traffic sources, and that they were not allowed to close the window;
- They were proposed to imagine themselves at home with friends or colleagues, and that behind the garden’s hedge, there was a crossroad and an industrial plant on the other side of the road;
- They were invited to perform a quiet and relaxing activity of their choice; examples of such activities were given: Reading, having a quiet conversation, playing cards; but they were not allowed to listen to music, give phone calls or watch videos.

Then, the experimenter went to the control room and played the first noise sequence. After each sequence, the participants were asked to answer a questionnaire. This questionnaire contained various questions:

- They were asked to write down the activity they performed during the exposure to the sound environment sequence;
- They had to assess how their activity was disturbed by the sound environment sequence on a continuous scale ranging from 0 “not at all disturbed” to 10 “extremely disturbed”;
- They had to answer the question whether they wished to close the window because of the sound environment sequence through a dichotomous choice (“yes” or “no”);
- They were asked to write which noise source they were able to identify during the sound environment sequence;
- They had to assess how much they were annoyed by the road traffic noise: “When you imagined yourself at home in presence of this sound environment sequence, did the road traffic noise annoy you?” (Question adapted from [10]). For this purpose, they were asked to use a continuous scale ranging from 0 “not at all annoyed” to 10 “extremely annoyed”;
- They had to assess how much they were annoyed by the industrial noise: “When you imagined yourself at home in presence of this sound environment sequence, did the industrial noise annoy you?” (Question adapted from [10]). They were asked to give their ratings along a continuous scale ranging from 0 “not at all annoyed” to 10 “extremely annoyed”;
- Finally, they had to assess how much they were annoyed by the overall sound environment sequence: “When you imagined yourself at home in presence of this sound environment sequence, did the overall noise, due to road traffic noise and industrial noise, annoy you?” (Question adapted from [10]). They were asked to answer on a continuous scale ranging from 0 “not at all annoyed” to 10 “extremely annoyed”.

At the end of the second part of the experiment, participants had to use three distinct continuous scales ranging from 0 “not at all to” 10 “extremely” in order to answer the following questions:

- “Would you say that you are sensitive to noise in general?”
- “Is for you a road infrastructure synonymous with danger?”
- “Is for you an industrial plant synonymous with danger?”

### 2.5. Participants

Fifty-one participants conducted the experiment (35 men, 16 women, mean age = 29, std. err. = 9.6 years). They were member staff and students from ENTPE recruited by email. They were told that we were looking for participants to a psychoacoustic test with relaxing activities in a dwelling room exposed to sound environment sequences with industrial and road traffic sources. All declared having normal hearing abilities. They were paid for their participation. Participants were separated in 12 groups of four or five participants each according to their availabilities.

### 2.6. Statistical Analysis

Statistical analyses, which included underlying hypotheses testing, were undertaken using STATISTICA software. First, correlation coefficients were calculated between annoyance ratings, noise sensitivity ratings and exposure noise levels in order to compare the results to findings from the *in situ* study [18,19] which combined noise situations were simulated in the current experiment. As the ratings were collected on a continuous scale during the experiment, Bravais-Pearson correlation coefficients r were calculated [27]. They are presented in Section 3.1.

Different analyses were carried out on different dependent variables collected during the experiment (e.g., activity disturbance, see [24] for further details). The analyses presented in Section 3 and Section 4 related to annoyance were carried out to fulfill the two aims of the experiment presented in this paper.

The first aim dealt with the potential effects on annoyance of some acoustical factors, non-acoustical factors and interactions between the combined noise sources under study. The studied acoustical factors related to the noise exposure were indoor L_ind_ and L_road_. The non-acoustical factors related to participants were the type of activity performed (denoted Ac), the noise sensitivity (denoted Se), the fear of danger from road infrastructure (denoted FR) and the fear of danger from industrial plant (denoted FI). The potential effects of these factors were studied on partial annoyance due to road traffic noise (denoted A_road_), on partial annoyance due to industrial noise (denoted A_ind_) and on total annoyance due to combined industrial and road traffic noises (denoted A_T_).

In order to investigate the effects of L_ind_ and L_road_ (and their interaction) on A_ind_, A_road_ and A_T_, 2-way repeated measures analyses of variance (RM ANOVA) were undertaken [27]. In order to investigate the effects of Ac, Se, FI and FR on A_ind_, A_road_ and A_T_, analyses of variance (ANOVA) [27] were carried out. All these analyses are summarized in Table 1. The results related to the first aim of the experiment are presented in Section 3.

**Table 1:** Summary of ANOVA carried out to fulfill the first aim of the experiment.

Annoyance	Independent Variables Under Consideration	
	Acoustical Factor	Non-Acoustical Factor
Partial industrial noise annoyance (A_ind_)	L_ind_	Fear from industrial plant (FI)
	L_road_	Noise Sensitivity (Se)
		Activity performed (Ac)
	
	2-way RM ANOVA	1-way ANOVA
Partial road traffic noise annoyance (A_road_)	L_ind_	Fear from road infrastructure (FR)
	L_road_	Noise Sensitivity (Se)
		Activity performed (Ac)
	
	2-way RM ANOVA	1-way ANOVA
Total annoyance (A_T_)	L_ind_	Fear from road infrastructure (FR)
	L_road_	Fear from industrial plant (FI)
		Noise Sensitivity (Se)
		Activity performed (Ac)
	
	2-way RM ANOVA	1-way ANOVA

The second aim of this work was to assess the goodness-of-fit of total annoyance models and their prediction power using data collected *in situ* [18,19,20]. To fulfil this aim, linear regression analyses were undertaken (*cf*. Section 4).

## 3. Effects of Independent Variables on Annoyance

The homogeneous character of the ratings collected during each part of the experiment was checked using Student’s t-tests on responses collected for the four common stimuli used in both parts of the experiment (*cf*. Section 2.4). As no significant differences were found in those responses collected in each part of the experiment, the data collected during both parts of the experiment were analyzed together.

### 3.1. Correlation Analyses

L_ind_ was significantly correlated with A_ind_ and A_T_ (respectively *r* = 0.27, *p* < 0.001; *r* = 0.1, *p* < 0.005). L_road_ was significantly correlated with A_road_ and A_T_ (respectively *r* = 0.33, *p* < 0.001; *r* = 0.30, *p* < 0.001). A_road_, A_ind_ and A_T_ were significantly correlated with Se (r ranged from 0.24 to 0.38).

### 3.2. Effects of Acoustical Factors

#### 3.2.1. On Partial Annoyance due to Industrial Noise—A_ind_

The 2-way RM ANOVA showed that L_ind_ factor had a significant effect on partial annoyance A_ind_ due to industrial noise [F(3,150) = 44.59; *p* < 0.001]. According to the effect size measure η^2^ (*i.e.*, the proportion of variance explained by this factor [27]), the effect was moderate (η^2^ = 9%). The L_road_ factor had no significant effect on A_ind_ [F(3,150) = 1.32; n.s. (*i.e*., non-significant)]. Thus the increase in road traffic noise level had no influence on the annoyance due to industrial noise when heard within the combined sequence. In other words, the partial annoyance due to industrial noise was not influenced by the presence of road traffic noise.

#### 3.2.2. On Partial Annoyance due to Road Traffic Noise—A_road_

The 2-way RM ANOVA highlighted that L_road_ factor had a significant effect on partial annoyance A_road_ due to road traffic noise [F(3,150) = 61.48; *p* < 0.001] with a moderate effect size measure (η^2^ = 11%). The L_ind_ factor had no significant effect on A_road_ [F(3,150) = 0.41; n.s.]. Thus a similar conclusion as the one concerning A_ind_ could be drawn: The partial annoyance due to road traffic noise was not influenced by the presence of industrial noise.

#### 3.2.3. On Total Annoyance due to Combined Industrial and Road Traffic Noises—A_T_

The 2-way RM ANOVA showed that both factors L_road_ and L_ind_ had a significant effect on total annoyance A_T_ with moderate and weak effect size measure respectively ([F(3,150) = 51.88; *p* < 0.001; η^2^ = 9%] and [F(3,150) = 9.17; *p* < 0.001; η^2^ = 2%] respectively). These results showed that the road traffic noise had a stronger effect on total noise annoyance in comparison with the industrial noise. This was due to the high difference in noise levels between these combined noises. But the results also showed that for the noise level range under consideration, the industrial noise had a significant weak influence on total annoyance. This was explained by the intermittent character of the road traffic noise and the steady character of industrial noise which was present between urban road vehicle pass-bys (*cf*. Figure 1).

**Figure 3:**
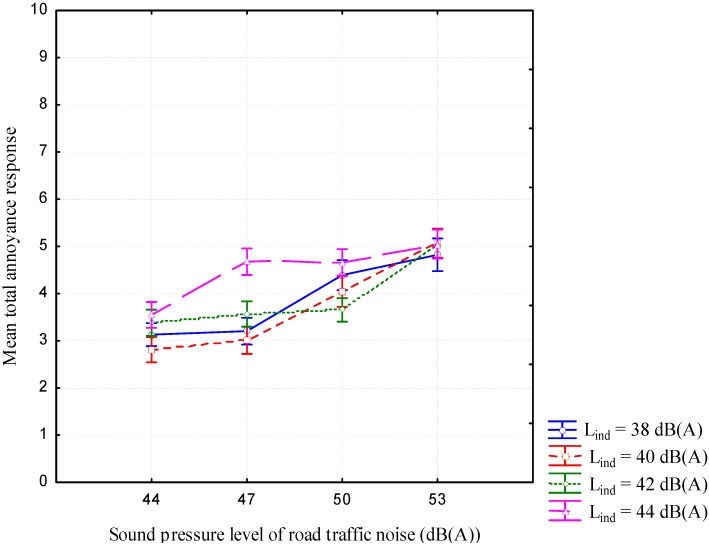
Mean total annoyance responses in function of factor L_road_ and categorized by each level of factor L_ind_. Error bars represent the standard error.

The 2-way RM ANOVA also highlighted a significant effect on total annoyance of the interaction between the two factors with weak effect size measure [F(9,450) = 3.14; *p* < 0.001; η^2^ = 2%]. Figure 3 illustrates this interaction.

The effect on total annoyance of the interaction between the combined noise source levels was due to the fact that at the higher level of industrial noise equal to 44 dB (A), the total annoyance did not increase anymore when the road traffic noise level increased from 47 dB (A) up to 53 dB (A).

### 3.3. Effects of Non-acoustical Factors

The activity performed by participants during each noise sequence and gathered after each noise sequence belonged to one of these four activity types: conversation (42% of the performed activities), individual concentration such as reading or crossword puzzle (32% of the performed activities), party game such as card game or jackstraws (25% of the performed activities) or just having a rest (only 1% of the performed activities).

The 1-way ANOVA (*cf*. Table 1) highlighted that each independent variable had a significant effect on the dependent variable under consideration. Table 2 details the significant effect of each main factor for each 1-way ANOVA performed. One can notice that values of the effect size measure η^2^ were different. Among all the studied independent variables related to non-acoustical factors, the activity performed (Ac) explained the least amount of variance observed in the different dependent variables (η^2^ was ranged from 2% to 9% according to the dependent variable under consideration). The fear of danger from industrial plant (FI) explained between 8% and 13% of the variance. Then the fear of danger from road infrastructure (FR) explained between 10% and 15% of the variance. Finally, noise sensitivity (Se) explained the greatest amount of variance observed in the different dependent variables (η^2^ was ranged from 10% to 21%).

**Table 2:** 1-way ANOVA results.

Annoyance	Independent Variable	Degree of Freedom	F	*p*-Value	η^2^ Effect Size Measure
A_ind_	FI	9	6.52	<0.001	0.08
	Se	10	8.51	<0.001	0.10
	Ac	3	5.87	<0.001	0.02
A_road_	FR	11	8.43	<0.001	0.10
	Se	10	17.87	<0.001	0.18
	Ac	3	25.80	<0.001	0.09
A_T_	FI	9	13.21	<0.001	0.13
	FR	11	12.67	<0.001	0.15
	Se	10	21.49	<0.001	0.21
	Ac	3	20.12	<0.001	0.07

A_ind_: Partial annoyance due to industrial noise; A_road_: Partial annoyance due to road traffic noise; A_T_: Total annoyance; FI: Fear of danger from industrial plant; FR: Fear of danger from road infrastructure; Se: Noise sensitivity; Ac: Activity performed.

**Figure 4:**
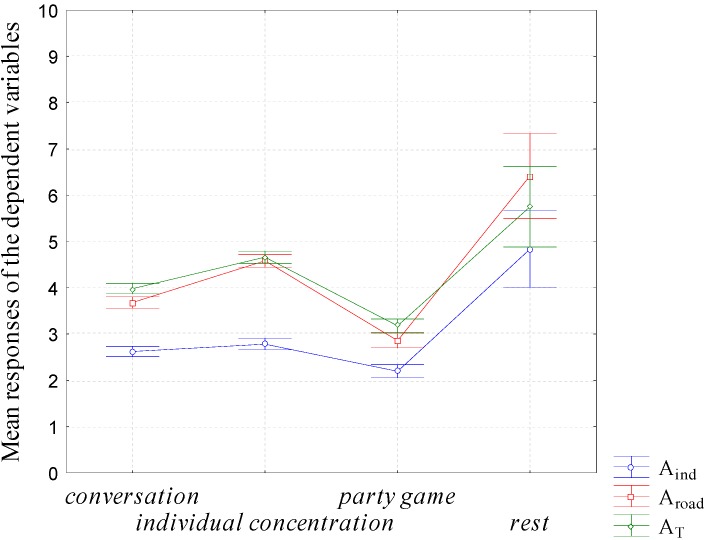
Mean responses of the dependent variables *vs.* the type of activity (Ac). Error bars represent the standard error. A_ind_: Partial annoyance due to industrial noise. A_road_: Partial annoyance due to road traffic noise. A_T_: Total annoyance.

As already mentioned, the 1-way ANOVAs showed that the activity performed (Ac) explained the least amount of variance (between 2% and 9% according to the dependent variable under consideration) but it was important to analyze the different quiet activities proposed to the participants in terms of their different influence on the reported noise annoyance. For each annoyance dependent variable, Tukey’s HSD test [27] revealed that participants having a rest were more annoyed during the experiment than participants playing a party game. The Tukey’s HSD test also revealed that except for partial annoyance due to industrial noise, participants playing a party game were less annoyed than participants having a conversation. The participants having a conversation were less annoyed than participants performing an individual activity. Figure 4 displays the mean responses of the dependent variables *versus* the activity performed by the participants. Due the weak proportion of participants having a rest, the standard error for this activity was the greatest.

### 3.4. Comparison of Partial Annoyance and Total Annoyance

In order to investigate potential interactions between the two combined noise sources, related to perceptual phenomena (e.g., paradox effect [6]), and comparison of partial and total annoyances was performed using paired *t*-tests.

These comparisons showed that total annoyance was generally close to the highest partial annoyance. Actually for only one combination among the 16 under study, a synergetic effect was observed, *i.e.*, the total annoyance was significantly higher than the highest partial annoyance. For the other 15 combinations studied, there was no significant difference between the total annoyance and the highest partial annoyance. Thus, a strongest component phenomenon was mainly highlighted. It could then be expected that the strongest component model proposed in literature [28] would be appropriate to predict the total annoyance due to the combined industrial and road traffic noises. Comparison of the two partial annoyances showed that the strongest component was mainly the partial annoyance due to the road traffic noise (A_road_ was superior to A_ind_ in 14 combinations on 16).

## 4. Total Annoyance Model Assessment

### 4.1. The Models

The second aim of the experiment concerned the assessment of total annoyance models from literature: (1) in terms of their goodness-of-fit when linear regression models were built from the data collected during the simulated environment experiment, and (2) in terms of their predictive power using *in situ* collected data during a survey [18,19,20].

The total annoyance models were selected according to the independent variables available from the survey [18,19,20] in order to assess their predictive power. These *in situ* data were partial and total annoyance ratings and the values of the day-evening-night level L_den_ for each single noise exposure and for the global noise due to the combined noise sources [18,19,20]. The annoyance data were collected from 99 respondents [18] (50 men and 49 women; mean age = 45.9 years with SD = 17.9; length of residence = 11.6 years with SD = 10.3; Working 45.5%; 8% worked or had a relative who worked on the industrial site). The annoyance ratings available [20] for testing annoyance models were gathered using the questions recommended by the ISO standards [10] and a continuous scale with two numerical labels 0 and 10 at both ends [18]. Eight total annoyance models from the literature were assessed (see for example [7,18,19] for an extensive description and discussion of these models):

− Four psychophysical models using independent variables constructed from L_Aeq_ (*cf*. Table 3):
  ○ Energy summation model [29], based on the overall sound pressure level (L_T_) calculated as an energy summation of L_Aeq_ of each combined noise;
  ○ Energy difference model [29], based on the overall sound pressure level (L_T_) and the absolute difference between L_Aeq_ of each combined noise to account for possible interaction;
  ○ Independent effect model [29], based on L_Aeq_ of each combined noise;
  ○ Weighted summation model [30], based on the total rating sound level (Lt) defined by:
    (1) $$L_{t}=k.log\left(10^{\frac{L_{ref}}{k}}+10^{\frac{L_{1}+P_{1}}{k}}\right)$$
    where k is a parameter that optimizes the model, L_ref_ is the L_Aeq_ of the source chosen as the reference source in the combination, L_1_ is the L_Aeq_ of the other source of the combination, and P_1_ the parameter calculated to obtain the equally annoying source reference level and given by:
    (2) $$P_{1}=\frac{b_{1}-b_{ref}+\left(a_{1}-a_{ref}\right)L_{1}}{a_{ref}}$$
    where a_ref_, b_ref_, a_1_ and b_1_ were coefficients of linear regressions between L_Aeq_ of each combined noise source and their respective annoyance:
    (3) $$A_{ref}=a_{ref}L_{ref}+b_{ref}$$
    (4) $$A_{1}=a_{1}L_{1}+b_{1}$$
− Four perceptual models using independent variables constructed from mean partial annoyance ratings (*cf*. Table 3):
  ○ Strongest component model [28];
  ○ Vector summation model [28] using an angle α that may allow to account for interaction and to optimize the model;
  ○ Linear regression model [31];
  ○ Mixed model [18,19] based on the absolute difference between the partial annoyances to account for possible interaction.

### 4.2. Goodness-of-Fit of the Total Annoyance Models Built from the Experiment Data

Table 3 summarizes the results of regression analyses performed for the assessment of the total annoyance models in terms of goodness-of-fit. The models were built linear regression analyses carried out between total annoyance responses (A_T_) collected during the simulated environment experiment, and the experiment independent variables (L_Aeq_ for psychophysical models and mean partial annoyances for perceptual models, respectively). The higher the determination coefficient (R^2^) and the lower the standard error of the estimate (std. err.), the better the goodness-of-fit.

**Table 3:** Total annoyance model assessment in terms of goodness-of-fit.

	Model	Regression Equation	R^2^	Std.err.
Psychophysical models	Energy summation	A_T_ = 0.25L_T_^d^ − 8.12	0.82	0.35
	Energy difference	A_T_ = 0.30*(1.08)*L_T_^d^ − 0.05*(−0.22)|*L_road_-L_ind_|^a^ − 9.82	0.82	0.34
	Independent effects	A_T_ = 0.22*(0.85)*L_road_^d^ + 0.08*(0.21)*L_ind_^a^ − 10.36	0.80	0.36
	Weighted summation (*k* = 7)	A_T_ = 0.31L_t_^d^ − 10.24	0.84	0.33
Perceptual models	Strongest component	A_T_ = 0.93max(A_road_,A_ind_)^d^ + 0.44	0.88	0.29
	Vector summation (α = 106°)	A_T_ = 1.05 √(A_road_^2^ + A_ind_^2^ + 2A_road_A_ind_cosα)^d^ − 0.18	0.99	0.10
	Linear regression	A_T_ = 0.81*(0.85)*A_road_^d^ + 0.48*(0.41)*A_ind_^d^ − 0.29	0.98	0.13
	Mixed	A_T_ = 0.64*(0.67)*A_road_^d^ + 0.62*(0.53)*A_ind_^d^ + 0.19*(0.21)|*A_road_-A_ind_|^a^ − 0.27	0.98	0.12

R^2^: The determination coefficient. Std. err.: The standard error of the estimate. Numbers in italics between brackets correspond to the standardized regression coefficient. ^a^ n.s. ^b^ *p* < 0.05. ^c^ *p* < 0.01. ^d^ *p* < 0.001. L_road_: The A-weighted equivalent sound pressure level of the road traffic noise. L_ind_: The A-weighted equivalent sound pressure level of the industrial noise. L_T_: The A-weighted overall sound pressure level. L_t_: Total rating sound level calculated with the parameter k [30]. A_ind_: Partial annoyance due to industrial noise; A_road_: Partial annoyance due to road traffic noise. A_T_: Total annoyance.

Two models involve the calculation of specific parameters. For the weighted summation model [30], the industrial noise was considered as the reference source, and the best goodness-of-fit was obtained with the parameter k equal to 7 for the calculation of the total rating sound level L_t_. For the vector summation model [28], an angle α = 106° allowed the model to be optimized.

**Figure 5:**
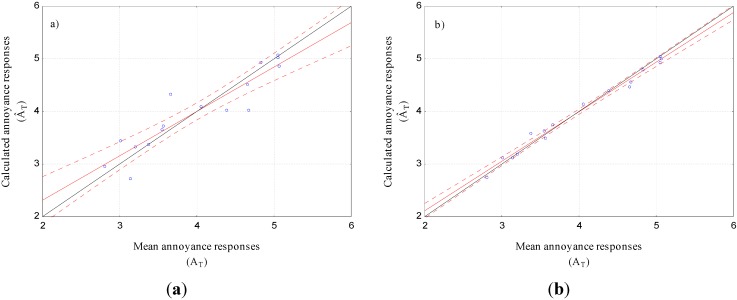
Calculated total annoyance responses (Â_T_) *versus* mean annoyance responses collected during the simulated environment experiment (A_T_); (**a**) the weighted summation model (k = 7); (**b**) the vector summation model (α = 106°).

Concerning the psychophysical models, Table 3 showed that the weighted summation model led to the best calculation of total annoyance, although the differences with the other psychophysical models were small. Figure 5 shows that the weighted summation model was a good model with no tendency to overestimate or underestimate total annoyance, even if the spread of data points appeared generally large.

Concerning the perceptual models, Table 3 shows that the strongest component model yielded the worst calculation of total annoyance, whereas the other three models better succeeded. The result relative to the strongest component model was not expected as the strongest component phenomenon was mainly highlighted for the different combined noise situations studied (*cf.* Section 3.4).

Furthermore, Table 3 shows that perceptual models provided a better calculation of total annoyance than psychophysical models. This result is illustrated by Figure 5 displaying the best psychophysical model (the weighted summation model), and the best perceptual model (the vector summation model). As a matter of fact, the spread of data points in the scatter plot concerning the vector summation model was smaller than the one concerning the weighted summation model.

### 4.3. Total Annoyance Model Testing Using Data Collected in Situ

The three best models highlighted in the previous section (*i.e.*, the three perceptual models with the best goodness-of-fit: The vector summation model, the linear regression model and the mixed model) were considered to be assessed in terms of their predictive power. The partial annoyance responses A_road_ and A_ind_, collected during the survey [18,19,20], were used in the equations built from the data collected during the simulated environment experiment (*cf.* Table 3). Thus predicted annoyance responses were obtained (Â_T_’).

The predictive power of these equations was assessed using r, a and b: r is the Bravais-Pearson coefficient correlation between A_T_ (total annoyance collected *in situ*) and Â_T_’ (predicted total annoyance), a and b are the slope and intercept of the regression line between A_T_ and Â_T_’.

**Figure 6:**
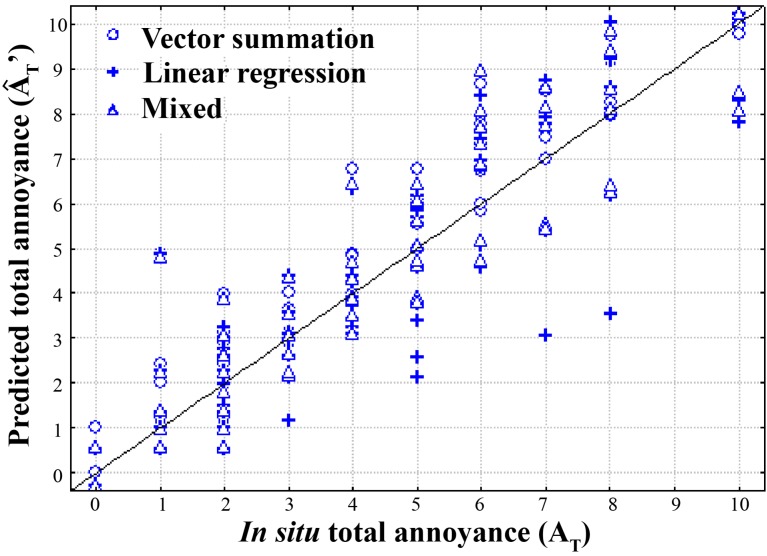
Scatter plot representing the results of total annoyance model testing.

Table 4 and Figure 6 show that the vector summation and mixed models performed best, and led to quite similar results. In Figure 6, data points were more closely clustered to the regression line using the vector summation and the mixed model relative to the linear regression model.

**Table 4:** Total annoyance model testing results.

Model	r ^d^	a	b
Vector summation	0.96	1.02	0.47
Linear regression	0.86	0.92	0.26
Mixed model	0.92	0.99	0.23

**^d^** *p* < 0.001.

## 5. Discussion

The correlation analyses carried out on the data collected during the experiment highlighted significant correlation coefficients between:

- Road traffic noise level and partial road traffic noise annoyance (*r* = 0.33, *p* < 0.001),
- Industrial noise level and partial industrial noise annoyance (*r* = 0.27, *p* < 0.001),
- Annoyances and noise sensitivity collected on 1-item scale (r ranged from 0.24 to 0.38).

The results obtained for annoyance due to road traffic noise were in agreement with those of different field studies (e.g., [13,32]), in terms of either correlation coefficient between annoyance and noise level, or correlation coefficient between annoyance and noise sensitivity. Furthermore, the simulated environment experiment dealing with annoyance due to combined industrial and road traffic noises allowed to provide results in agreement with the findings of the socio-acoustic survey [18,19] from which the current experiment was based on. Actually, Pierrette *et al.* [18,19] found a significant correlation coefficient, equal to 0.38, between road traffic noise level and partial road traffic noise annoyance. They found a significant correlation coefficient, equal to 0.27, between industrial noise level and partial industrial noise annoyance. Pierrette *et al.* also found significant correlation coefficient between annoyances and noise sensitivity, the correlation coefficients were ranged from 0.2 to 0.27 [18,19].

Concerning the influence of the activity performed during the experiment on annoyance responses, we found similar results as the one highlighted by Öhrström *et al.* [32] in their field study dealing with road traffic noise exposure: Activities involving speech communication were less impacted by noise compared to concentration and relaxation activities. Thus, the experiment simulated real-life situations and combined noise exposures allowed to provide results in agreement with findings of field studies.

The ANOVA carried out in the current work showed that the exposure to road traffic noise had no significant effect on partial annoyance due to the industrial noise, and that the exposure to industrial noise had no significant effect on partial annoyance due to road traffic noise. Thus the partial annoyances were not influenced by the presence of the other noise in the combination, contrary to inhibition observed between combined transportation noises (e.g., [4]). One possible explanation lies in the fact that for the considered noise sequences, the differences in sound pressure level between the highly varying road traffic noise and the steady and continuous industrial noise were important. Due to these differences, road vehicle noises, when present, masked almost completely the industrial noise. This masking effect had also been observed *in situ* for these two combined noises [18,19] and led to a description of the steady industrial noise as an intermittent noise by the residents as they only heard it when no road traffic pass-by noise events were present.

The 2-way RM ANOVA showed the strongest effect of L_road_ on the total annoyance as it was expected, but this ANOVA also showed that despite the relatively important differences in L_Aeq_ between the two combined noises, significant weak effects of L_ind_ and of the interaction between L_road_ and L_ind_ existed on total annoyance A_T_.

The comparison of total annoyance and partial annoyances highlighted mainly a strongest component phenomenon, and occasionally but significantly, a synergetic effect. These results were in agreement with the significant standardized regression coefficients assigned to variables based on A_road_ and A_ind_ in the perceptual models (*cf.* Table 3). These results were also in agreement with observations carried out *in situ* by Pierrette *et al.* [18,19]: Despite a lower level of the industrial noise in the combination, the corresponding partial annoyance was non-negligible as only 51% of people surveyed found the industrial noise less annoying than the road traffic noise, and 27% of them found both noises equally annoying.

The comparison of total annoyance models (*cf. *Table 3 and Figure 5) showed that perceptual models are superior to psychophysical models concerning total annoyance calculation, which was congruent with literature findings (e.g., [18,19]). Moreover, the results presented in Table 3 pointed out that the strongest component model was not the best model whereas a strongest component phenomenon was mainly highlighted for the studied combined noise situations. Both *in situ* and laboratory noise annoyance studies had also pointed out such limitations of this model [7,18,19,31]. The main drawbacks of the strongest component model lie in the fact that it does not allow to take into account (1) possible influence of variations in the partial annoyance of the least annoying noise on the total annoyance [31], and (2) possible interactions between combined noises [7,18,19]. This result was confirmed in the experiment using important differences in noise levels between the two combined noises which mainly led to the strongest component phenomenon.

The total annoyance model testing consisted in injecting the data of an *in situ* survey into the model equations built from the simulated environment experiment. The comparison of perceptual models highlighted that the vector summation and the mixed models allowed a better prediction of *in situ* total annoyance than the linear regression model (*cf. *Table 4 and Figure 6). This result was congruent with results obtained by Pierrette *et al.* [18,19]. This showed that it was necessary to propose models allowing possible interactions between noises to be taken into account (the angle α used in the vector summation model and the absolute difference term used in the mixed model were examples of such possibility).

This work pointed out that the simulated environment experiment was useful to contribute to enhance total noise annoyance model. Actually, this experiment which simulated combined industrial and road traffic noises observed *in situ* allowed the construction of perceptual total annoyance models with a good predictive power.

This work also highlighted the limit due to *in situ* data available for model testing*.* Actually no annoyance models including both acoustical and non-acoustical variables (e.g., multilevel regression model using acoustical variables and noise sensitivity [33]) were built and tested in this work. This suggests encouraging surveys which allow to collect *in situ* data available for model testing. Such surveys might be carried out with common procedures in order to collect annoyance and influential non-acoustical factors on continuous 1-item scale as recently done by Ecotière *et al*. [34] for a survey dealing with transportation noise. 

Such suggestions would be useful to improve the actual dose-effect relationships, used to model the impact on health of noise exposure in terms of noise annoyance, which is a challenge identified by WHO [2]. Furthermore, adequate dose-effect relationships used in a perceptual model of total annoyance could thus help noise action planners, especially in urban areas where inhabitants are exposed to multiple noise sources. As a matter of fact, the European Environment Agency [35] in its guideline recommends to use a psychophysical model based on actual dose-effects relationships which drawbacks are clearly identified [2].

## 6. Conclusions

This work dealt with total annoyance due to industrial and road traffic noises using a simulated environment experiment. During the experiment, participants were in conditions approaching real-life situation. They were exposed to combined industrial and road traffic noise sequences that simulated the noise environment of a suburban city of Lyon where a socio-acoustic survey was carried out.

The main results were the following:

− Concerning acoustical factors, no inhibition effect on partial annoyance due to the presence of the other noise were highlighted. Also, due to the difference in sound pressure levels between the two combined noises, the road traffic noise level had a greater influence on total annoyance than the industrial noise level;
− All of the considered non-acoustical factors had significant effects on the studied dependent variables;
− The comparison of partial and total annoyances mainly highlighted a strongest component effect;
− The assessment and comparison of total annoyance models confirmed the superiority of perceptual models over psychophysical models;
− The testing of the total annoyance models using *in situ* data highlighted the superiority of the perceptual models with interaction term. Such result is interesting as such models allow interaction between combined noises to be taken into account even in the studied case of total annoyance mainly governed by strongest component phenomenon.

Thus, these results reinforced the need to focus on perceptual models and to improve the prediction of partial annoyances. On the long term, this could also help to improve noise action planning especially in urban areas where multiple noise source environments are most often experienced by inhabitants.

## Supplementary Files

Supplementary File 1
