# Supplementary material for: A Simulated Environment Experiment on Annoyance Due to Combined Road Traffic and Industrial Noises
Source: Int J Environ Res Public Health. 2015 Jul 21;12(7):8413–33. doi: 10.3390/ijerph120708413 (PMC4515728; doi:10.3390/ijerph120708413)
Supplement: Supplementary File 1 [file ijerph-12-08413-s001.pdf]

Supplementary Information

A Simulated Environment Experiment on Annoyance due to Combined Road Traffic and Industrial Noises

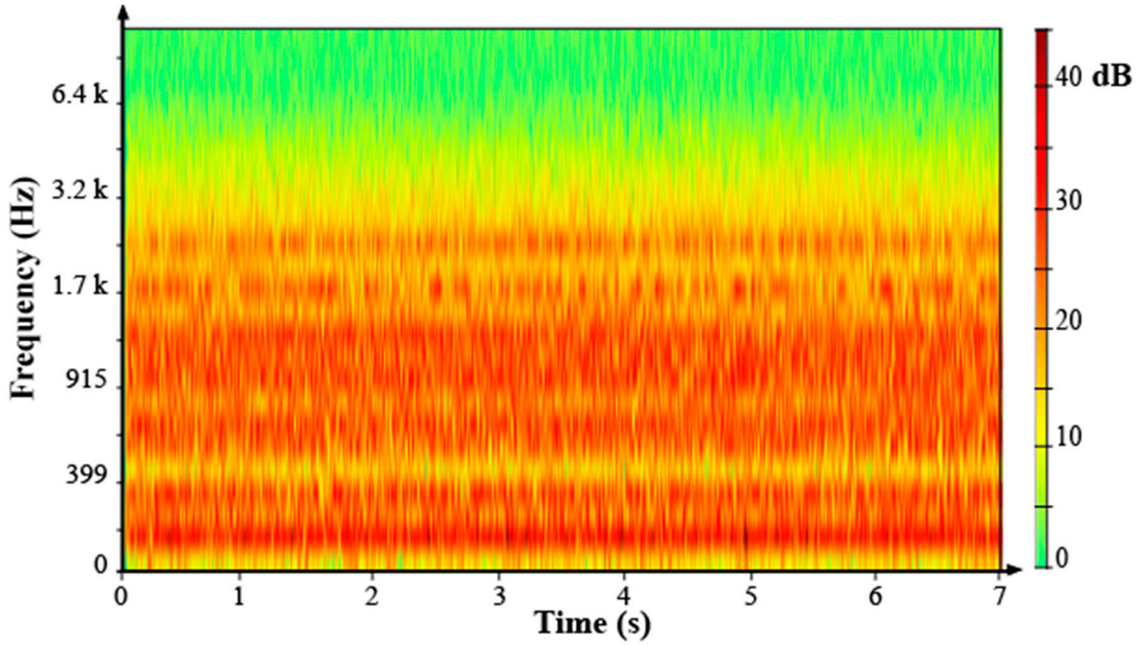

Figure S1. Spectrogram for an excerpt (7s) of the studied stationary industrial noise: zoom on its frequency content.

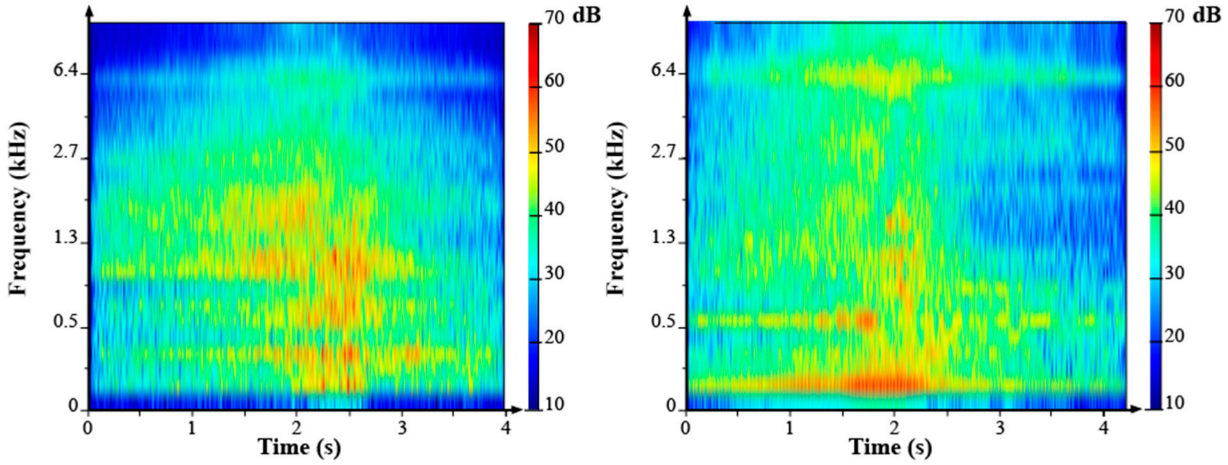

Figure S2. *Cont.*

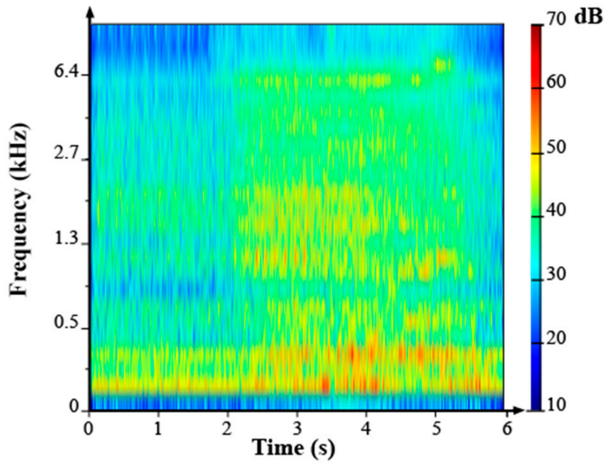

Figure S2. Spectrogram for excerpts of the studied urban road traffic noise: zoom on the spectral content due to urban road vehicle pass-by (a light vehicle at constant speed: top left, a powered-two-wheeler in deceleration: top right, a heavy vehicle in acceleration: bottom left).

© 2015 by the authors; licensee MDPI, Basel, Switzerland. This article is an open access article distributed under the terms and conditions of the Creative Commons Attribution license (http://creativecommons.org/licenses/by/4.0/).
